# Clinical and Electroencephalography Assessment of the Effects of Brivaracetam in the Treatment of Drug-Resistant Focal Epilepsy

**DOI:** 10.7759/cureus.15012

**Published:** 2021-05-13

**Authors:** Ersilia Savastano, Patrizia Pulitano, Maria Teresa Faedda, Leonardo Davì, Nicola Vanacore, Oriano Mecarelli

**Affiliations:** 1 UOC Neurologia, Ospedale Santo Bono-Pausilipon, Napoli, ITA; 2 UOC Neurofisiopatologia, Policlinico Umberto I, Rome, ITA; 3 Department of Human Neuroscience, Neurophysiopathology Unit, Policlinico Umberto I, Rome, ITA; 4 CNAPS Department (Promotion and Evaluation of Chronic Disease Prevention Policies), Istituto Superiore di Sanità (ISS), Rome, ITA; 5 Department of Human Neuroscience, Neurophysiopathology Unit, Policlinico Umberto I, University of Rome “Sapienza”, Rome, ITA

**Keywords:** brivaracetam, levetiracetam, focal drug resistant epilepsy, quantitative eeg, pharmaco-eeg, neurocognitive tests

## Abstract

Introduction: Our aim was to evaluate the clinical and electroencephalographic effects of brivaracetam (BRV) in patients with drug-resistant focal epilepsy. BRV is a new antiepileptic drug (AED) with a high affinity for vesicle protein 2A (SV2A) and recently approved as adjunctive therapy for focal onset seizures. Methods: In this observational study of six-month duration, BRV (50-200 mg) was administered to 76 patients with drug-resistant focal epilepsy, who were ≥16-year-old and who suffered from daily, weekly, monthly and yearly recurrent seizures. At baseline and after six months of follow-up, we performed a neurological visit, neuropsychological tests: Quality of life in epilepsy-31 (QOLIE31), Epworth Sleepiness Scale (ESS), Intrapersonal Emotional Quotient (IEQ) and an electroencephalogram (EEG; inspective and quantitative analysis). Twenty-four patients underwent an overnight switch from levetiracetam (LEV) to BRV. Results: Seizure frequency of the 54 patients remaining at six months was reduced >50% in 29.6% of cases (responders), <50% in 31.5% (non-responders 1), while it remained unchanged in 38.8% (non-responders 2). Twenty-nine percent of patients early discontinued BRV because of lack of efficacy or minor adverse effects (AEs) like irritability, asthenia or headache. Neuropsychological tests in 28 patients demonstrated a significant improvement in I-EPI scores (p=0.04). Comparable results have been found in the subgroup of patients who switched from LEV to BRV. The EEG quantitative analysis showed a significant reduction of alpha absolute power at six months (p=0.03). Theta band power resulted significantly superior in non-responders than in responders (p=0.03). Furthermore, the δ+θ/α+β index resulted more elevated in patients with AEs than in patients without. Conclusions: BRV showed discrete results in terms of efficacy, safety and tolerability, with a good behavioural profile. BRV reduces the power of the alpha band, in correlation with its sedative effects but not with its minor efficacy. Furthermore, the increase in theta band power can be considered as a predictor of scarce response to treatment, while an increase in the δ+θ/α+β index could be a possible predictor of AEs occurrence.

## Introduction

Drug-resistant epilepsy is defined by the International League Against Epilepsy (ILAE) as the failure of adequate trials of two tolerated and appropriately chosen and used antiepileptic drugs (AEDs) schedules, whether as monotherapies or in combination, to achieve sustained seizure freedom [1]. This condition affects more than 30% of people with epilepsy, and in previous meta-analyses, an annual cumulative incidence of 67.7/100,000/year has been reported, with differences linked to socio-economical, geographical and demographical factors. The percentage of drug resistance between adults is double of that paediatric populations (30% vs 15%) and the risk of developing such condition is higher in patients suffering from focal epilepsy (up to 60%) than in those with generalized epilepsy (20%) [2]. Drug-resistant epilepsy is due to numerous factors. Some of them are linked to the patient and the disease itself, while others are related to the AEDs and their mechanism of action [3]. A higher expression of P-glycoproteins, codified by the multidrug resistance mutation 1 (MDR1) and multidrug resistance mutation 2 (MDR2) genes, on the cell membrane is a crucial mechanism in the development of drug resistance and numerous AEDs, particularly phenytoin, phenobarbital and benzodiazepines, are known to induce it [4]. Brivaracetam (BRV), the n-propyl analogue of levetiracetam (LEV), has been approved as adjunctive therapy for focal onset seizures and focal to bilateral tonic-clonic seizures, in adults and children ≥4 years old. BRV shares with LEV its principal mechanism of action, which is its reversible binding to the synaptic vesicle glycoprotein 2A (SV2A), though showing a 30 times higher affinity than LEV. Other studies suggested that BRV could have a role in the inhibition of voltage-gated sodium channels [5]. Furthermore, unlikely its precursor, it is not a substrate for MDR1 (Pgp 1), multidrug resistance-associated protein 1 (MRP1) and multidrug resistance-associated protein 1 (MRP2) transporters [6]. For its characteristics, this new AED could have superior results than LEV in the treatment of drug-resistant epilepsy [7]. The efficacy and tolerability of BRV as an adjunctive therapy have been demonstrated in randomized and controlled, double-blind, phase III pilot studies, conducted on patients aged 16 years or more, suffering from focal onset seizures that were not controlled with one or two preceding drugs [8-10]. Other post-marketing studies on patients with drug-resistant epilepsy have been conducted in order to evaluate the efficacy and safety of BRV, achieving good results in terms of treatment response rate (27-35%) and tolerability features [11]. Regarding the effect of BRV over the brain’s bioelectrical activity, which is detectable by means of the EEG, no studies have been conducted to date. Previous works suggest that studying the effects of AEDs on EEG recordings could be a valid option for monitoring both neurotoxicity and adverse effects (AEs) and predicting their efficacy [12].

## Materials and methods

We conducted an observational study enrolling 76 consecutive patients admitted from July 2018 to July 2019 to our Epilepsy Center who were 16-years-old or more and with a diagnosis of drug-resistant epilepsy with focal onset seizures. We recorded demographical and clinical features at baseline (t0) relating to sex, age, history of epilepsy (years); etiology of epilepsy (2017 ILAE classification system); seizure frequency (daily/multi-daily; weekly/multi-weekly; monthly/multi-monthly ≤4 months; annual/multi-annual ≤1 month); seizures type; number and type of AEDs tested and currently in use. BRV was prescribed at an initial dosage of 25 mg BID, subsequently titrated on the basis of individual needs to a maximum dosage of 200 mg per day. For patients who reported the absence of efficacy or intolerable AEs while on LEV or BRV therapy were started with a conversion ratio of 15:1 as proposed by Klein et al. [13]. We performed a neuropsychological assessment of those patients who did not have previous cognitive deficits, at baseline (t0) and after six months (t1) of therapy. The evaluation was composed of three questionnaires: Quality of Life in Epilepsy 31 (QOLIE31), Epworth Sleepiness Scale (ESS) and Irritability in Epilepsy Questionnaire (I-EPI) [14-16]. Regarding EEG qualitative and quantitative analysis, the following exclusion criteria were applied: a referred seizure during the previous 24 hours; any modification of AED therapy between baseline EEG and follow-up at six months; <5 minutes of recorded activity free from artefacts; any neuroactive drug exposure other than AEDs in the preceding 24 hours. The authors evaluated each patient’s EEG by confronting epileptiform discharges (entity, location and morphology) and background activity (frequency, amplitude, regularity and stability) at t0 and t1. After the evaluation, each patient was allocated to one of the following categories: 1 = no variations in EEG activity; 2 = better EEG activity (any reduction of epileptiform discharges and/or slow-wave background activity); 3 = worse EEG activity (any increase of epileptiform discharges and/or slow-wave background activity). EEG tracings were then used for off-line quantitative analysis by fast Fourier transform. Selected epochs were used for calculating absolute (µV2) in the various EEG frequency bands (delta = 1-3.5 Hz; theta = 4-7.5 Hz; alpha = 8-12.5 Hz; beta 1 = 13-19.5 Hz; and beta 2 = 20-29 Hz). The following indexes were also calculated: peak power frequency (PPF, Hz), main dominant frequency (MDF, Hz); (δ+θ)/(α+β) index (calculated with the ratio between the absolute power of slower frequency bands and that of higher frequency bands). Demographical and clinical features of the study population are expressed as numbers (percentage) and/or absolute values, means and standard deviation (SD). Drug efficacy was evaluated at t1, considering any decrease in seizure frequency and/or intensity since t0, reported by the patients as a minor duration and/or gravity of seizure signs and symptoms. Patients who experienced a >50% reduction of seizures were considered responders, while the others were considered as non-responders. Non-responders group was further divided in two groups: non-responders 1 (reduction of seizure frequency < 50%) and non-responders 2 (none seizure variation). Demographical and clinical features of responders versus non-responders and of patients with AEs versus those without, compared by Student t-test for continuous variables and chi-square test for categorical variables. BRV safety and tolerability were assessed by defining the percentage of patients who discontinued the drug or who experienced AEs at three and six months. AEs were reported in terms of absolute values and percentages. Neuropsychological test results were described in terms of score means and SD. Mean scores at t0 and at t1 were then compared for every single test. A t-test for independent samples was performed in order to assess any statistically significant difference of neuropsychological tests mean scores between responders and non-responders and between patients with AEs and those without. Data from EEG analysis were described in terms of absolute values and frequencies (percentage). For each variable considered for EEG spectral analysis, a mean of all recordings at t0 and t1 was calculated. A t-test for paired samples was used to compare EEG parametric means at t0 and those at t1. A Student t-test for independent samples was then used to compare EEG parametrical means between responders and non-responders at t0 and at t1. P-values inferior or equal to 5% were considered statistically significant.

## Results

Of 76 patients enrolled in our study, 41 (54%) were females, and the mean age was 42 ± 15 years, with no difference between males (43.8 ± 9.7 years) and females (40.9 ± 17.2 years). Mean epilepsy duration was 23.15 ± 13.2 years. 46% of patients had an underlying structural cause (21% malformative, 13.2% vascular, 6.6% post-traumatic, 3.9% postsurgical, 1.3% with a space-occupying lesion), 51.4% had an unknown cause and 2.6% had a genetic etiology. Regarding seizures type, 46 patients (60.5%) presented with the focal onset and focal to bilateral tonic-clonic seizures, 10 patients (13.2%) with focal onset aware seizures and 20 patients (26.3%) with focal onset impaired awareness seizures. At baseline (t0), seizure frequency was: −26.3% (20 patients) daily/daily recurrent; −26.3% (20 patients) weekly/weekly recurrent; −32.9% (25 patients) monthly/monthly recurrent (less than 4/month); −14.5% (11 patients) annual/annual recurrent (< 1 per month). Thirty-three patients (43.4 %) had already been on therapy with three or more drugs and the remaining 43 patients (56.6%) had already tried at least two drugs at optimal doses without benefit. During the study period, together with BRV, 22 patients were taking one other AED, 28 patients two AEDs, 18 patients three AEDs and 7 patients were on polytherapy with four AEDs. Carbamazepine (CBZ) was the most used AED in association with BRV (36.8%), followed by valproic acid (VPA) (32.9%) and lamotrigine (LTG) (31.6%). 34% of patients were on therapy with other sodium channel blockers (eslicarbazepine, ESL; lacosamide, LCM; oxcarbazepine OXC), while benzodiazepines (BDZ) and phenobarbital (PB) were used by 23.7% and 22.4% of patients, respectively. Other drugs sporadically used in our population were perampanel (PER), topiramate (TPM), gabapentin (GBP) and acetazolamide (AAZ; Table 1).

**Table 1:** Demographic and clinical characteristics in the study population (76 patients). *No gender significant differences. CBZ: carbamazepine, VPA: valpoic acid, LTG: lamotrigine, BDZ: benzodiazepine, PB: phenobarbital, TPM: topiramate, PER: perampanel, LCM: lacosamide, OXC: oxarbazepine, ZNS: zonisamide, ESL: eslicarbazepine, GBP: gabapentin, AAZ: acetazolamide.

Mean age (years)	42 ± 15*	
History of epilepsy (years)	23.15 ± 13.2*	
	n	%
Gender		
F	41	54
M	35	46
Etiology		
Structural	35	46
Malformative	16	21
Vascular	10	13.2
Traumatic	5	6.6
Post-neurosurgery	3	3.9
LOS	3	1.3
Genetic	2	2.6
Unknown origin	39	51.4
Seizure type		
Focal with awareness	10	13.2
Focal without awareness	20	26.3
Focal to bilateral	46	60.5
Frequency of seizures		
≥1/day	20	26.3
≥1/week	20	26.3
≥1/month (<4/month)	25	32.9
≥1/year (<1/month)	11	14.5
Previous AEDs		
>3	33	43.4
≤3	43	56.6
Concomitant AEDs		
1	22	29
2	28	37.8
3	18	24
4	7	9.2
Concomitant AEDs		
CBZ	28	36.8
VPA	25	32.9
LTG	24	31.6
BDZ	18	23.7
PB	17	22.4
TPM	5	6.6
PER	4	5.3
LCM	4	5.3
OXC	4	5.3
ZNS	3	4
ESL	3	4
GBP	2	2.6
AAZ	2	2.6

**Figure 1:**
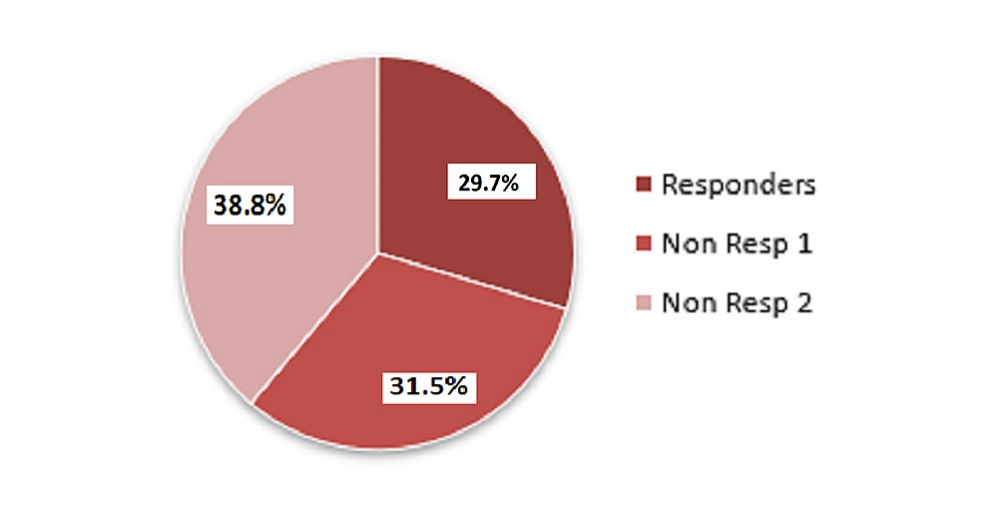
Response rate to brivaracetam (%). Responders: reduction in the frequency of seizures >50%, non-responders 1: reduction in the frequency of seizures < 50%, non-responders 2: unchanged frequency.

Of the 76 patients enrolled, 22 (29%) discontinued the study drug before six months follow-up. Of the remaining 54 patients, 16 patients (29.7%) were classified at six months as responders (seizure decrease >50%, with one patient classified as seizure-free), 17 patients (31.5%) as non-responders 1 (seizure decrease < 50%) and 21 patients (38.8%) as non-responders 2 (seizure frequency unchanged; Figure 1).

**Figure 2:**
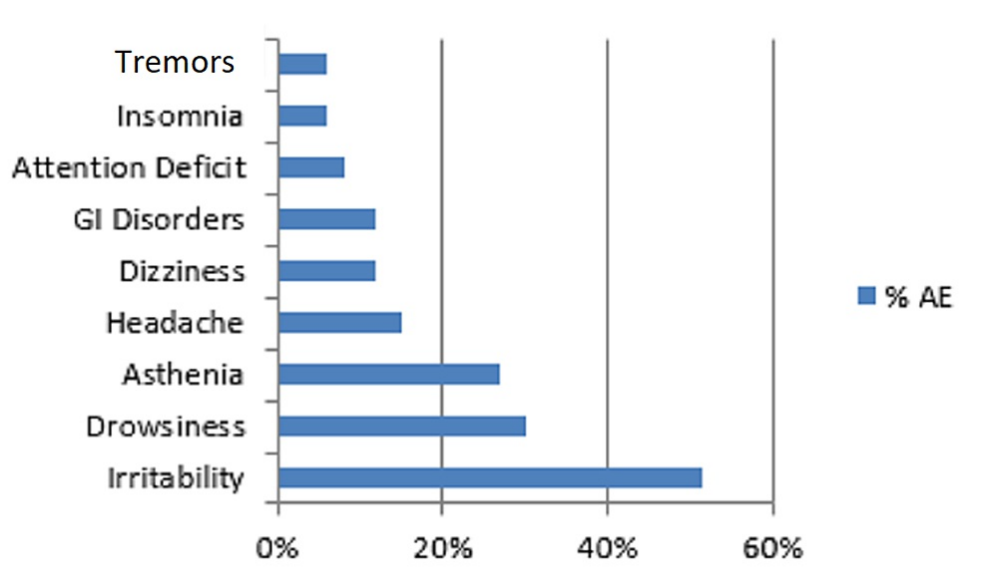
Frequency (%) of adverse effects in the patients (n=33) who referred them. GI: Gastrointestinal.

Regarding seizure frequency, six months after BRV was started, patients with daily and weekly recurrent seizures (n=19) decreased (35.1% vs 52.6%). When confronting responders and non-responders - 1/2, we found that patients with baseline daily recurrent seizures had a lower response to BRV therapy (p=0.025). Moreover, 33 patients (43.4%) reported at six months a subjective decrease of seizure intensity, defined as a reduction of seizure duration and gravity. Conversely, we did not find any statistically significant difference between responders and non-responders-1/2 regarding age (p=0.266), sex (p=0.175), epilepsy type (p=0.606), involved hemisphere (p=0.503), seizure type (p=0.505), epilepsy duration (p=0.955), number of previous and concomitant AEDs (p=0.557 and p=0.428, respectively). Mean dosage (+SD) of BRV was 131 + 38.3 mg per day in responders and 126.3 + 42.9 mg per day in non-responders - 1/2 (p=0.68). Particularly, of 16 responders, 6 were taking 100 mg per day, 7 were taking 150 mg per day and 3 patients were taking 200 mg per day. Among 16 responders, AEDs that were most frequently taken along with BRV were LCM (40%), LTG (33.3%) and TPM (20%). Contingency analysis did not show significant results in terms of BRV response when this was administrated in association with other drugs. Twenty-two of 76 patients enrolled (29%) early discontinued BRV, because of lack of efficacy in 3 patients (13.6%), AEs in 9 (40.9%) or both in 10 (45.5%). Thirty-three out of 76 patients (43%) reported AEs, of which the most frequent was irritability (52%). Other common AEs were somnolence (30%), asthenia (27%), headache (15%) and vertigo (12%; Figure 2).

**Figure 3:**
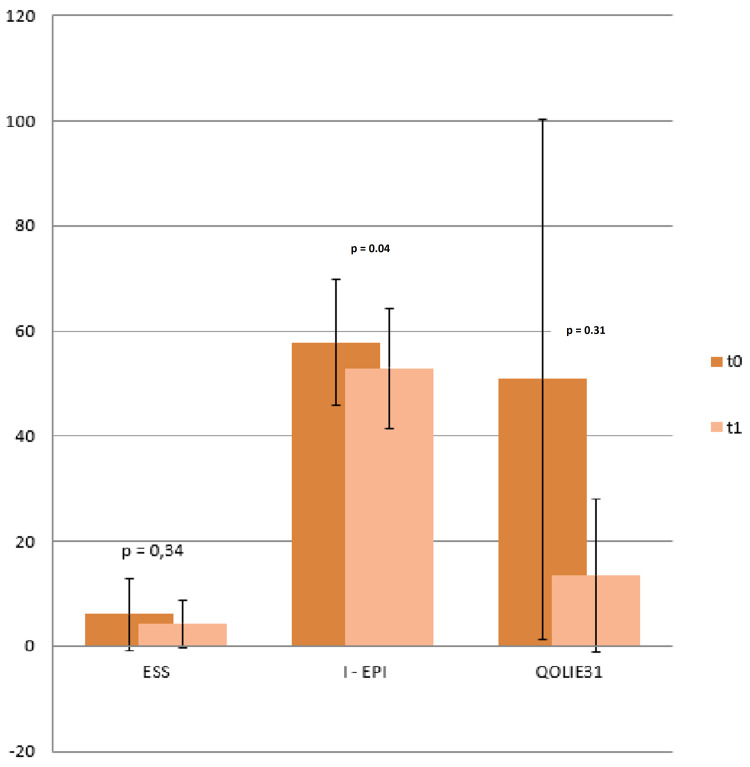
Average Score on ESS, I-EPI, QOLIE31 t0 vs t1. Student t-test showed a statistically significant improvement (p=0.04) only for the I-EPI score. ESS: Epworth Sleepiness Scale, I-EPI: Irritability in Epilepsy Questionnaire, QOLIE31: Quality of Life in Epilepsy 31.

A BRV dosage equal or superior to 150 mg per day was associated with a higher incidence of drug discontinuation because of AEs (p=0.015). However, we did not find any association between drug early discontinuation and other considered variables like age (p=0.431), sex (p=0.132), epilepsy cause (p=0.752), seizure type (p=0.299), involved hemisphere (p=0.254), number of concomitant drugs (p=0.098), epilepsy duration (p=0.344) and seizure frequency at baseline (p=0.098). Conversely, treatment with previous AEDs was significantly associated with early drug discontinuation, with a higher drop-out rate for those who were taking more than three drugs (p=0.019). Finally, the number of previous or concomitant AEDs was not associated with a higher degree of AEs development (p=0.093 and p=0.237, respectively) and contingency analysis did not show any statistically significant difference between relevant AEs and BRV association with CBZ (p=0.611) or VPA (p=0.459). A total of 37 patients underwent baseline and six months follow-up neuropsychological testing. When confronting mean scores at t0 and t1, we found a significant variation in I-EPI relative scores, with a reduction after BRV treatment (57.81 ± 12.35 at t0 vs 52.93 ± 11.37, p=0.039). On the other hand, we did not find a significant difference for other administered tests (ESS: t0 6.04±6.85 vs t 14.24 ± 4.51, p=0.34; QOLIE 31: t0 50.85 ± 13.45 vs t1 49.41 ± 14.49, p=0.31; Figure 3).

**Figure 4:**
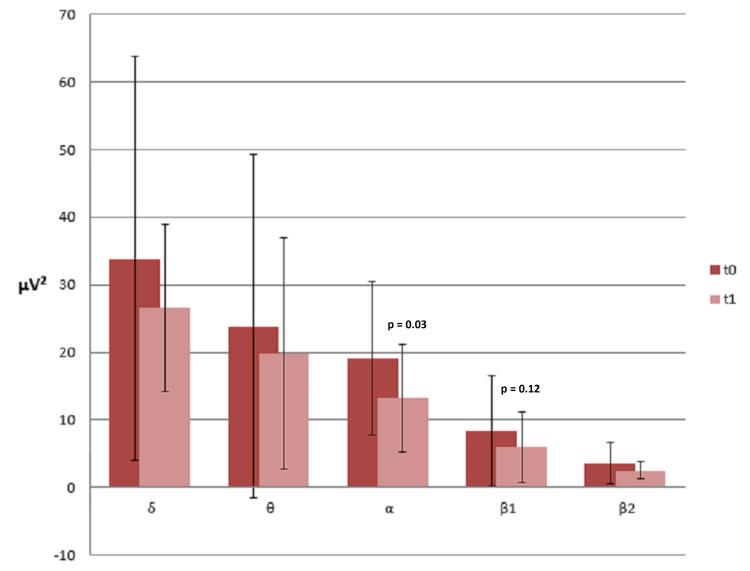
Absolute powers average electroencephalography t0 vs t1. From baseline to six months, the absolute power of alpha-band is reduced statistically significantly (to = 19.01 ± 11.37 μV2 vs t6 = 13.19 ± 7.98 μV2).

We selected 26 EEG recordings according to the methodology previously described. The inspective analysis did not show any relevant variation in epileptiform discharges between baseline evaluation and six months follow-up. At six months, in fact, 15 patients (57.6%) presented an unchanged EEG recording, 7 patients (26.9%) a better EEG recording and 4 patients (15.4%) a worse EEG recording. When a positive or negative variation was observed, it exclusively regarded the quantity of epileptiform discharges but not their site or morphology. About background activity, it was slower in 16 patients (61.5%) at six months, however, remaining in the same frequency range observed at baseline. Furthermore, we found greater stability and a higher trend in the diffuse transmission of background activity. In the other 10 patients, background activity remained unchanged. Regarding quantitative analysis, we herewith report the more relevant modifications. When confronting variables at baseline and at six months follow-up, we found that α absolute power was significantly reduced from a mean value of 19.01 ± 11.37 μV2 at t0 to a mean value of 13.19 ± 7.98 μV2 at t1 (p=0.03). We also found a trend towards reduction for β1 power, that passed from a mean value of 8.42 ± 8.1 μV2 at t0 to a mean value of 5.97 ± 5.17 μV2 at t1 (p=0.12; Figure 4).

**Figure 5:**
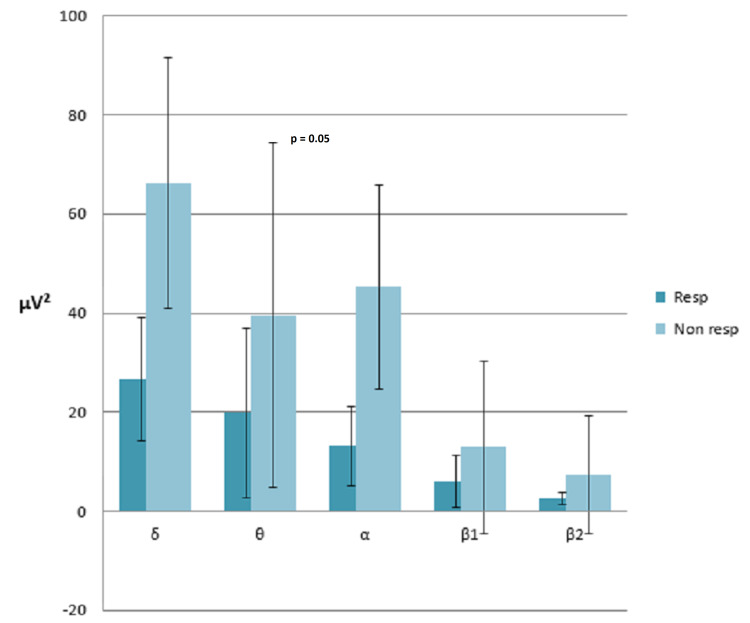
Average absolute powers band electroencephalography responders vs non-responders.

T-test for independent samples showed that at t0, responders and non-responders-1/2 significantly differed in terms of α power (19 ± 11.37μV2 vs 42 ± 48.4 μV2; p=0.041). However, at t1, this difference was not evident anymore, while θ power differed significantly, being inferior in responders than non-responders-1/2 (19.8 ± 17.1525 μV2 vs 45.3 ± 20.54μV2, p=0.058; Figure 5).

Comparing EEG recordings of patients who did not report AEs and those of patients with AEs, we found that the latter had an increment of d+q/a+b index (1.39 ± 1.09 vs 2.9 ± 2.34, p=0.05). Twenty-three patients underwent an overnight switch from LEV to BRV. Statistical analysis did not evidence significant differences at baseline between switch and non-switch patients regarding mean age (p=0.81), sex (p=0.31), epilepsy duration (p=0.55), epilepsy cause (p=0.10), seizure type (p=0.10), seizure frequency at baseline (p=0.15) and number of concomitant AEDs (p=0.44). After six months, four patients (16.7%) early discontinued the drug. Of the remaining 19 patients, 4 (21.1%) presented a seizure decrease >50%, 8 (42%) presented a seizure decrease <50% and 7 (36.8%) did not show any variation. Statistical analysis did not show any difference in terms of treatment response for the switch and non-switch patients (p=0.27). However, we found a seizure decrease in 21.1% of switch patients, but this reduction did not appear to be statistically significant (p=0.29). Four out of 23 switch patients (17.3%) discontinued treatment with BRV, one patient, because of lack of efficacy, one because of AEs and two because of both previous reasons. No difference was found between the switch and non-switch patients regarding discontinuation rate (p=0.15) and its cause (p=0.67). Thirteen out of 23 patients (56.5%) reported AEs, of which the most common were irritability (5), vertigo (4), insomnia (3), asthenia (3), somnolence (1), headache (1), tremor (1), gastrointestinal discomfort (1). No difference was found between the switch and non-switch patients (p=0.51). Neuropsychological evaluation at t0 and t1 was performed in 12 switch patients. When confronting scores at t0 and t1, we observed a significant reduction of I-EPI score (54.75 ± 12.42 vs 45 ± 9.244; p=0.01). We did not evidence any significant difference between switch and non-switch patients at t0 in terms of testing results, while at t1 we observed a trend towards significance in I-EPI score (t 1 p=0.07 vs t0 p=0.23). We selected seven EEG recordings of switch patients for qualitative and quantitative analysis. However, they did not show any significant results.

## Discussion

The population of our study presents typical features of drug-resistant epilepsy (The International League Against Epilepsy (ILAE) defines drug-resistant epilepsy as “failure of adequate trials of two tolerated, appropriately chosen and used AED schedules (whether as monotherapies or in combination) to achieve sustained seizure freedom) [2,3]. A structural etiology is found in almost half of our patients; disease mean duration is high (23.15 ± 13.2 years) and the number of previous AEDs was superior to >3 in 44% and <3 in 56% of patients. Furthermore, epilepsy severity in our population is highlighted by the presence of focal onset to bilateral tonic-clonic seizures (60.5% of patients) and by the high frequency of seizures themselves. All of these factors are in fact markers of epilepsy severity and are positively associated with drug resistance. Seizure recurrence would determine modifications in neuronal connectivity, neurotransmitters imbalance and dysfunction and altered neuronal metabolism [17,18]. Finally, during the study period, almost 80% of patients were taking two or more AEDs together with BRV (predominantly VPA, CBZ and LTG), according to pharmacological strategies normally used in patients with drug-resistant epilepsy. Excluding drop-out patients, of the remaining 54 patients, 16 of them (29.9%) were considered responders, while 17 (31.5%) were non-responders 1, and 21 (38.7%) non-responders 2. In double-blind randomized and placebo-controlled trials evaluating patients with focal onset seizures that were poorly controlled by drug therapy, a BRV dosage of 100 mg per day led to a responders percentage ranging from 30% to 36% [10]. In a retrospective multicenter study conducted by Villanueva et al. [18] on 575 patients with focal onset epilepsy (25% with focal to bilateral seizures, seizure mean frequency 17/month), after one year of BRV therapy (range dose 25-400 mg) a percentage of 17.5% were seizure-free and of 39.7% were classifiable as responders [19]. If considering the limits of the present study and its less numerous study population, our result, however relevant, seems inferior. This could be due to higher drug resistance and higher seizure frequency at baseline in our patients, being this last variable associated with a lower response to BRV. However, similar results are reported by Steinhoff et al. [19] in one real-life experience on 101 patients with drug-resistant focal epilepsy treated with BRV. In this monocentric survey, the authors found after three months of treatment that 27.8% of the patients were responders and 7% seizure-free [20]. Regarding BRV dosage, we did not find a significant difference in terms of treatment response from 75 to 200 mg per day and this is in line with previous meta-analyses. Among the 16 patients considered responders, the more frequent concomitant AEDs were LCM (40%), LTG (33.3%) and TPM (20%). This is in line with previous data showing a synergistic effect between BRV and LTG or TPM without a significant increase of AEs [21]. However, our observation is driven by a low number of patients in single drug combination groups, especially that of BRV plus TPM (n=4). Regarding BRV tolerability in our population, 29% of patients early discontinued BRV therapy, mostly because of AEs but also because of lack of efficacy. Cognitive-behavioural AEs (irritability, somnolence and asthenia) were the most frequent and this is similar for other new AEDs. A number of more than three previous AEDs were predictive of early BRV discontinuation. This variable is in fact a marker of epilepsy severity and of treatment response. It is also possible that previous failing therapeutic strategies with other AEDs could have induced in these patients fear of unexpected AEs and distrust for new pharmacological treatments, leading to subjective early discontinuation of the drug. In our population, 43% of patients presented AEs, the most frequent being irritability, somnolence, asthenia, headache and vertigo, with results comparable to those already described in other studies [22]. Irritability and somnolence are AEs frequently encountered during therapy with LEV, the precursor of BRV, while vertigo seems to be more often reported by patients treated with BRV. This could be due to its higher affinity to SV2A vesicles or to higher levels of CBZ-epoxide when association therapy of BRV and CBZ is used. However, previous data did not highlight particular recommendations regarding the association between BRV and CBZ, confirming that BRV can be safely used with other AEDs [23]. Regarding behavioural disorders, the I-EPI test showed in our patients a significant decrease of irritability after six months of BRV therapy, and this result likely confirms that of another neuropsychological study conducted on 43 patients, which considered irritability and aggressiveness [24]. However, our study evidences a divergence between I-EPI scores and AEs subjectively reported by the patients, the most common of which was irritability. These conflicting data could be explained as a study bias caused by the fact that, in order to confront the behavioural effects of BRV and LEV, irritability was assessed with specific questions that could have suggested in some ways the answer to the patient. Another explanation could be that cognitive-behavioural effects are in fact hardly quantifiable subjective experiences. EEG quantitative analysis showed after six months a decrease in α and β1 absolute powers and this result correlates well with those of the inspective analysis, that is a slowing in background activity described in more than half of EEG recordings at six months. To the best of our knowledge, this is the first work on EEG spectral analysis of the BRV effect, but we can compare our results to those of other AEDs. While BDZs and barbiturates at low-medium doses determine an increase in rapid bands power (beta1 and beta2), all the other AEDs can cause a slowing in the dominant rhythm (alfa), a reduction of the power of more rapid bands (similarly to what we described for BRV) and/or an increase of slow theta and delta activities [25]. Previous studies suggest that pharmaco-EEG could be a reliable means to determine AEDs neurotoxicity (i.e., cognitive disorders, asthenia, somnolence, etc.), even at early stages of drug treatment. More generally, it could be considered as an objective neurophysiological method by which we can foresee the efficacy and tolerability of AEDs. Pharmaco-EEG guidelines support the electroencephalographic study of drugs, saying that it can be applied to those purposes any time a drug has detectable effects over the central nervous system (CNS) [26,27]. Mecarelli et al. demonstrated that the modifications of cognitive activity in epileptic patients during and after AED therapy discontinuation correlated well with the peak of alpha rhythm frequency [28]. For example, when analyzing the EEG modifications induced by the AEDs, CBZ can cause a slowing of alpha background activity and an increased power of theta and delta bands. Those modifications, which also depend on plasma concentration, have been related to its sedative effects and they do not compromise its efficacy. Those EEG modifications are typical of other sodium and calcium blocking agents like OXC and GBP. A reduction in α power is also induced by TPM and it was related to its sedative and neurocognitive effects [29]. Finally, a selective increase in theta and gamma frequencies has been demonstrated for ESL and associated with better brain connectivity. In our study, BRV determined a reduction of alpha power after six months of treatment, acting like TPM, and this could explain its similar, however, lower, sedative and neurocognitive effects. The EEG quantitative study was effectuated first on the entire study population at t0 and t6; then, it was also performed by confronting responders and non-responders. This evaluation highlighted a statistically significant difference in theta band absolute power, which was higher in non-responders. This could be a useful neurophysiological marker to predict BRV efficacy. Finally, when we compared the numerical index obtained by the delta+theta/alfa+beta ratio in patients with and without AEs, we found that it is higher in the subgroup of patients who reported AEs (predominantly somnolence and asthenia), reflecting a higher electrical activity slowing and suggesting that this index could be used as a marker of neurotoxicity. In our study, 46% of patients switched from LEV to BRV because of the lack of efficacy of the former. Klein’s hypothesis supports that, in case of lack of efficacy of LEV, the probability of a response to BRV is low [29]. We did not confirm that observation because the patients who underwent a pharmacological switch showed efficacy outcomes comparable to those patients who did not perform any switch. Numerous post-marketing studies confirm our results, concluding that switching to BRV when LEV is not effective is reasonable [11]. Our patients switched from LEV to BRV because of AEs, mostly behavioural, in 54% of cases. In this subgroup of patients, 16.6% early discontinued BRV while 58.3% reported treatment-emergent AEs already described. When confronting switch and non-switch groups regarding treatment-emergent AEs, we did not find any statistically significant difference. Furthermore, the results of neuropsychological tests showed a significant amelioration of I-EPI scores among switch patients after six months of BRV treatment. Other researches support our results, highlighting a decrease in sedation and non-psychotic behavioural disorders associated with LEV among switch patients and suggesting that a pharmacological switch in those patients presenting with behavioural AEs may be reasonable [25,30]. On the contrary, Yates et al. tried to describe the neurobehavioural effects of LEV and BRV, obtaining EEG and neuropsychological quantitative measurements and concluding that the cognitive and electrophysiological effects of BRV were similar to those of LEV. The scarce tolerability of LEV would not lead to a higher risk of BRV behavioural disorders, probably because the latter does not interact with AMPA receptors and has a more selective effect on SV2A.

## Conclusions

In our study, we enrolled a population of epileptic patients who presented baseline clinical features suggestive of disease gravity, like a long disease duration, focal onset to bilateral tonic-clonic seizures with weekly and/or daily recurrent frequency and a scarce response to previous numerous pharmacological therapies. Although its efficacy was partially and negatively influenced by the gravity markers that we have just described, BRV showed a discrete efficacy, mostly among patients with a seizure frequency less pronounced. Furthermore, we observed a better efficacy profile when BRV was associated with LTG and TPM. Its tolerability profile was also good, as we did not report serious AEs during the six months of treatment, but only minor AEs with an early discontinuation rate that was not inferior to that of other newer AEDs. Considering our results, we can also conclude that BRV has an acceptable cognitive-behavioural profile; despite irritability has been one of the more frequently reported AEs, this has not been confirmed by neuropsychological tests that, on the contrary, showed better results in this context. Either inspective or quantitative EEG analyses showed a background activity slowing after BRV, which correlated well with the sedative effects frequently reported in our population. Furthermore, the quantitative analysis highlighted an increased theta band’s absolute power which, in our opinion, could be a predictive marker of lack of efficacy of BRV, while the increase of delta+theta/alfa+beta index could be considered as a predictor of neurotoxicity. The switch from LEV to BRV, whether for lack of efficacy or for AEs, appeared as a feasible strategy. Indeed, our results permit us to conclude that a previous exposition to LEV is not predictive of lack of efficacy or a higher risk of AEs when switching to BRV. Moreover, LEV-naïve patients and patients who switched from LEV did not differ regarding their response to BRV and their tolerability to the new treatment. We can reasonably conclude that when it is necessary to suspend LEV because of AEs which are mostly behavioural, it is advisable to switch to BRV because it reduces those complications.
